# A Case Series on Post-COVID Tuberculosis: An Underrated Duo of COVID-19 and Tuberculosis

**DOI:** 10.7759/cureus.48013

**Published:** 2023-10-30

**Authors:** Jayakumar Rajagopal, Sandeep Konaka Gautamdas, Gayathri Sivakumar, Karthikeyan Ramaraju

**Affiliations:** 1 Respiratory Medicine, PSG Institute of Medical Sciences and Research, Coimbatore, IND; 2 Pharmacy, PSG College of Pharmacy, Coimbatore, IND

**Keywords:** adolescent tuberculosis, post-covid-19 complications, disseminated tuberculosis, post-covid tuberculosis, tuberculosis

## Abstract

In India, tuberculosis (TB) has the second highest disease burden following diabetes mellitus. During the COVID-19 pandemic, there was a surge of several opportunistic infections. In this case series, we report five patients, including three adults and two adolescents, who have developed various forms of TB disease after symptomatic COVID-19 pneumonia. The average time for development of post-COVID TB was 48 days. Adolescent patients have developed disseminated TB, which can be due to COVID-19-induced immunological injury or its treatment-related immune suppression. All the adult patients had high CT severity scores (CTSS) and required the administration of intravenous steroids during their COVID-19 pneumonia. Various presentations of TB were secondary spontaneous pneumothorax, miliary TB, consolidation, and nodular infiltrates. One patient had a drug-induced liver injury, which complicated the treatment of that patient. Factors that may contribute to the development of post-COVID TB are diabetes mellitus, increased severity of COVID-19 pneumonia manifested by CTSS, and administration of intravenous steroids. Bidirectional screening of TB had to be done when patients present with symptoms of COVID-19 pneumonia.

## Introduction

Tuberculosis (TB) is an infectious disease caused by the *Mycobacterium tuberculosis complex *affecting the lungs and other body parts such as the brain, spine, and kidney [1]. According to the World Health Organization, globally around 10.6 million people were affected with TB, and 1.6 million deaths were reported in 2021. Globally, an increase of 4.5% in TB cases was seen during 2021 compared to 2020 [2]. In India, the total number of TB cases reported during 2021 was 19% higher compared to 2020 [3]. Severe acute respiratory syndrome coronavirus 2 (SARS-CoV-2), also known as coronavirus disease 2019 (COVID-19), created a disastrous impact on global health by affecting many millions of people and causing death in higher numbers. During the COVID-19 pandemic period, many opportunistic infections such as candidiasis, mucormycosis, aspergillosis, cryptococcosis, Pneumocystis pneumonia, TB, and viral infections (*Cytomegalovirus*, herpes simplex virus) were reported [4]. During the COVID-19 pandemic period in India, there was a drop in the detection of TB compared to the pre-pandemic period [5]. According to WHO, in India, there was a 2.8% decline in TB detection and an 11% increase in mortality due to TB during 2020 compared to the previous year [6]. There were case reports on TB after COVID-19 recovery from all over the world, including the United States of America, Turkey, Bangladesh, Mexico, Iran, Saudi Arabia, Qatar, Morocco, Pakistan, Sri Lanka, and South Africa. Tadolini M et al. reported 14 cases of TB as post-COVID sequelae, which were developed after a median of four days [7]. This case series aims to describe patients who developed TB following COVID-19 in a tertiary care hospital.

## Case presentation

Case 1

A 54-year-old male who was a known diabetic, hypertensive, and had ischemic heart disease presented with complaints of non-productive cough, breathlessness, and fever. The patient was diagnosed with type 1 respiratory failure and tested positive for COVID-19 three months ago during which he had a CT severity score of 17/25 and was treated with intravenous (IV) steroids and antivirals, as shown in Table 1. During the current admission, saturation was 98%, requiring 2 liters of oxygen. Laboratory investigations showed evidence of uncontrolled diabetes mellitus where glycated hemoglobin (HbA1c) was found to be 10.7%, as shown in Table 2. Sputum culture was positive for the growth of methicillin-resistant *Staphylococcus aureus*, which was community-acquired. Chest X-ray showed non-homogenous opacity in the left lower zone, suggesting collapse with consolidation. Due to uncontrolled diabetes, a bronchoscopy was done to rule out lower lung field TB, which showed purulent secretions in the left main bronchus, which was edematous and narrowed. Bronchoalveolar lavage (BAL) was taken and sent for an acid-fast bacilli (AFB) smear, which turned out to be negative but the GeneXpert detected rifampicin-sensitive *Mycobacterium tuberculosis*. The patient was initiated on an anti-tuberculosis treatment (ATT) regimen according to the weight band and discharged. The patient was followed up till the completion of the regimen. No adverse reactions or non-adherence was found.

**Table 1:** Characteristics of patients admitted during COVID-19 RT-PCR: real-time reverse transcription polymerase chain reaction test (performed for detection of COVID-19); CTSS: computed tomography severity score.

Case	Age	Sex	Comorbidities	RT-PCR report	CTSS	Treatment given during COVID-19	
						Oxygen supplementation	Steroid
1	54	M	Diabetes mellitus, hypertension, ischemic heart disease, type 1 respiratory failure	Positive	17/25	Yes	Yes
2	15	F	Nil	Positive	0/25	No	No
3	17	F	Nil	Positive	0/25	No	No
4	61	M	Diabetes mellitus, hypertension, coronary artery disease, hypothyroidism	Positive	13/25	Yes	Yes
5	67	F	Diabetes mellitus, hypertension	Positive	15/25	Yes	Yes

**Table 2:** Characteristics of patients during post-COVID tuberculosis * Number of days between COVID-19 infection and development of tuberculosis; NA: not applicable.

Case	No. of days*	Glycated hemoglobin (%)	Microbiological confirmation	Radiological confirmation	Complication
1	76	10.7	Bronchoalveolar lavage	Bronchiectasis	Nil
2	20	NA	Clinically diagnosed	Miliary nodules	Anti-tuberculosis treatment-induced hepatitis
3	18	NA	Sputum	Miliary nodules with tree-in-bud bronchiolitis	Nil
4	57	7.7	Sputum	Nodular consolidation	Nil
5	71	7.9	Bronchoalveolar lavage	Consolidation with cavities, and left hydropneumothorax	Nil

Case 2

**Figure 1:**
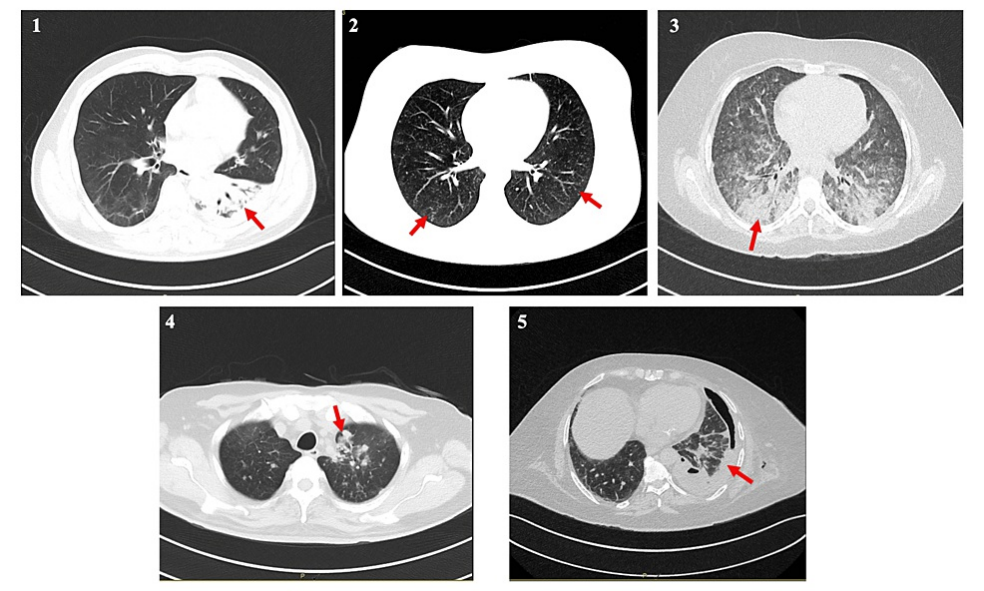
High-resolution computed tomography scan of the patients after developing post-COVID tuberculosis Case 1: Dense consolidation of left lower lobe. Case 2: Diffuse miliary nodules. Case 3: Patchy infiltrates present in both lower lobes. Case 4: Nodular infiltrates present in the left upper lobe. Case 5: Left lower cavitary consolidation complicated with left hydropneumothorax.

A 15-year-old female with no known comorbidities tested positive for COVID-19 infection 20 days back, during which a CT chest was done, which showed a normal lung with prominent mediastinal nodes with an ill-defined iso-dense lesion adjacent to the pancreatic head. The patient was treated conservatively and advised to follow up after one week for evaluation of the iso-dense lesion but the patient did not turn up. During the second admission, high-resolution CT (HRCT) of the lungs showed diffuse miliary nodules in both lung fields in random distribution and a few enlarged mediastinal nodes in lower paratracheal and subcarinal regions with matting and calcification, as shown in Figure 1. Sputum GeneXpert was negative for *Mycobacterium tuberculosis*. Contrast-enhanced computed tomography of the abdomen showed conglomerate necrotic nodes in the portocaval region posterior to and indenting the head of the pancreas. Endoscopic ultrasound-guided fine needle aspiration cytology from the portocaval node showed necrotizing granulomatous inflammation suggestive of TB. Retinal examination showed a placoid lesion in the supratemporal quadrant of the right eye (choroid tubercle). The patient was diagnosed with disseminated TB and was initiated on ATT based on her weight. After three weeks of initiation of ATT, she was readmitted with nausea, vomiting, and abdominal pain. Her liver enzymes were elevated and she was not tolerating the conventional first-line drugs and hence was initiated on modified ATT. She was followed up and had a good symptomatic recovery and gained 8 kg over six months.

Case 3

A 17-year-old female with no known comorbidities was treated for COVID-19 infection. Laboratory investigations showed elevated inflammatory markers, D-dimer, and a decreased level of hemoglobin (9.2 g/dl), suggestive of anemia. HRCT showed a COVID-19 Reporting and Data System (CO-RADS) grade 6 and CT severity score was 0/25. She was treated symptomatically and discharged. After 19 days, she was readmitted with complaints of fever, breathlessness, and bleeding per rectum. Her saturation was 84% in room air and 95% with 2 liters of oxygen. Lab investigations showed serial elevation of interleukin-6 (45.29 pg/ml, 69.75 pg/ml, and 70.27 pg/ml) and D-dimer (2.18 mg/ml, 2.25 mg/ml, and 5.93 mg/ml). The CT pulmonary angiography showed features of atypical viral pneumonia with tiny random miliary nodules with tree-in-bud bronchiolitis. Ultrasound of the neck showed multiple necrotic cervical lymph nodes. Examination of the retina showed choroid tubercles. Sputum GeneXpert detected the *Mycobacterium tuberculosis complex*. The patient was diagnosed with disseminated TB and was treated with ATT. The patient was followed up over six months and improved symptomatically with a weight gain of 5 kg over six months.

Case 4

A 61-year-old male who was known diabetic, hypertensive, and had hypothyroid and coronary artery disease (CAD) was admitted in November 2020 with complaints of generalized tiredness, loss of appetite, and sore throat for two days and tested positive for COVID-19. The saturation was at 96% with the requirement of 8 liters of oxygen. Laboratory investigations showed elevated inflammatory markers, such as lactate dehydrogenase (LDH) (745 U/L), ferritin (1178 ng/ml), interleukin-6 (67.66 pg/ml), troponin T (1506 pg/ml), D-dimer (0.86 mg/L), and erythrocyte sedimentation rate (ESR) (30 mm). The patient was treated with antiviral drug remdesivir for five days and IV steroids for 10 days. During the management of COVID-19, diabetes mellitus was fairly controlled by the administration of metformin 500 mg twice daily along with the insulin sliding scale. Eventually, the patient became symptomatically better and was discharged. After two months, the patient was readmitted with complaints of breathlessness for seven days. During the present admission, his saturation was at 98% with a requirement of 2 liters of oxygen. Laboratory investigations showed elevated HbA1c (7.7%), indicating uncontrolled diabetes mellitus. CT of pulmonary angiography showed patchy areas of consolidation with multiple small nodules causing tree-in-bud appearance in the apical-posterior segments of both upper lobes and superior segments of both lower lobes, extensive ground glass haziness with traction bronchiectasis in both lower lobes, suggesting sequelae of COVID-19 pneumonia with secondary infection, and cardiomegaly. Sputum GeneXpert detected the *Mycobacterium tuberculosis complex*. He was started on the ATT regimen. After six months of completion of ATT, the patient became symptomatically better.

Case 5

A 67-year-old female who was known diabetic and hypertensive presented with complaints of chest pain, cough, and breathlessness for 15 days duration. She was treated for COVID-19 two months ago. Her CT severity score was 15/25 and she was treated with antivirals and IV steroids with oxygen supplementation for two days. She improved symptomatically and was discharged without oxygen. During the present admission, saturation was 95% on room air with reduced breath sounds in the left infrascapular area. Baseline investigations showed an elevated HbA1c (7.9%). Chest X-ray revealed an air-fluid level in the left mid and lower zone suggesting large left-sided hydropneumothorax. Under strict aseptic precautions, a 28F intercostal drainage tube was inserted in the left 5th intercostal space in the anterior axillary line. Pleural fluid was exudative with adenosine de-aminase of 56 U/L. Pleural fluid cytology revealed acute dense inflammation and GeneXpert was negative. The bacterial culture of the fluid turned out to be sterile. Contrast-enhanced computed tomography of the chest showed patchy areas of collapse consolidation with cavities in the left lower lobe and moderate left hydropneumothorax with multiple loculations with mildly enlarged subcarinal lymph nodes, as shown in Figure 1. Bronchoscopy showed right and left upper lobe bronchus to be edematous with no endobronchial lesions or extrinsic compression. Lavage was taken from the bilateral upper lobes and left lower lobe. BAL GeneXpert detected rifampicin-sensitive *Mycobacterium tuberculosis*. The patient was initiated on a fixed drug combination for drug-sensitive TB. After three days, a chest X-ray was repeated, which showed an unexpanded lung on the left side. The chest drain was then connected to negative suction of -20 cm H2O, following which there was adequate re-expansion. The patient was followed up till the completion of ATT, no adverse reactions were found and the patient became symptomatically better.

## Discussion

In this case series, five patients, including three adults and two adolescents, had developed TB as post-COVID sequelae. The time for the development of active TB disease after COVID-19 was found to be 48.8 ± 27.2 days where patients managed without steroids developed earlier (19 ± 1.4 days) compared to the patients treated with steroids (68 ± 9.8 days). Among the five patients, two of the adolescents developed disseminated TB. BCG vaccination protects children up to 15 years of age against severe forms of TB, after which adolescents are susceptible to TB disease [8]. The incidence of disseminated TB is higher in adolescents compared to the adult population [9]. Therefore, the adolescent population is at a higher risk for developing disseminated TB due to COVID-19-induced immunosuppression. Adult patients had high CT severity scores and were treated with IV steroids. During COVID-19, decreased counts of CD4+ T-cells and CD8+ T-cells (lower than 300 cells/µL and 400 cells/µL, respectively) were reported [10]. T-cells provide immunity against *Mycobacterium tuberculosis*, which is compromised in COVID-19. The diabetic patients in this case series had poor glycemic control, which can be attributed to COVID-19 and also the use of steroids as a part of COVID-19 treatment. The SARS-CoV-2 virus attaches to the angiotensin-converting enzyme type 2 (ACE-2) receptors expressed in the pancreas based on severity, and it can lead to new-onset diabetes and poor control in diabetic patients after COVID-19. Interferon-γ and tumor necrosis factor-α are the two vital cytokines involved in preventing *Mycobacterium tuberculosis* infection that are reduced in patients with diabetes mellitus [11]. The cytokine dysregulation in diabetic patients causes impaired immune responses against *Mycobacterium tuberculosis*, resulting in active TB infection. The Ministry of Health and Family Welfare of India has published guidelines on bidirectional screening where TB should be screened in COVID-19 patients who experience cough, fever, or breathlessness for more than two weeks [12]. Various presentations of TB identified in this case series include bronchiectasis, miliary TB with dissemination, and pneumothorax. The challenge faced in the treatment of TB was drug-induced liver injury (DILI), where one among the five patients developed DILI. The incidence of ATT-induced hepatitis in the pre-COVID period was found to be 3% [13]. SARS-CoV-2 attaches to the ACE-2 receptors expressed in the liver (2.6%) and bile duct (59.7%), which explains the possible mechanism for liver damage during COVID-19 infection [14]. In severe COVID-19 infection, hypoxia, acute respiratory distress syndrome, and cardiac failure can occur, which can cause hepatic ischemia leading to hepatic inflammation, which explains the higher incidence of acute hepatic injury following initiation of anti-tuberculous treatment [15].

## Conclusions

In this case series, five patients developed TB disease after COVID-19 pneumonia with an average time of 48 days. This emphasizes the fact that patients infected with the COVID-19 virus are prone to develop some forms of active TB disease due to alterations in pulmonary and systemic immunological defense mechanisms. Bidirectional screening for TB and COVID-19 should be followed for patients who present with COVID-19 symptoms. Active screening for TB disease during the first few months of follow-up is also recommended. Irrespective of age, gender, history of contact, and vaccination status, TB should be anticipated as one of the treatable complications of COVID-19 pneumonia. Comorbidity of diabetes mellitus, increased severity of COVID-19 pneumonia manifested by CT severity score, and administration of parenteral or enteral steroids may be associated with increased frequency in the incidence of TB. Further research is required to support this observation.
